# Genetic diversity of *Plasmodium Vivax* revealed by the merozoite surface protein-1 icb5-6 fragment

**DOI:** 10.1186/s40249-017-0302-6

**Published:** 2017-06-05

**Authors:** Wei Ruan, Ling-ling Zhang, Yan Feng, Xuan Zhang, Hua-liang Chen, Qiao-yi Lu, Li-nong Yao, Wei Hu

**Affiliations:** 1 Department of Communicable Diseases of Control and Prevention, Zhejiang Provincial Centre for Disease Control and Prevention, Hangzhou, China; 2 School of Life Sciences, FuDan University, Shanghai, China

**Keywords:** *Plasmodium vivax*, *PvMSP-1*, Genetic diversity, Malaria

## Abstract

**Background:**

*Plasmodium vivax* remains a potential cause of morbidity and mortality for people living in its endemic areas. Understanding the genetic diversity of *P. vivax* from different regions is valuable for studying population dynamics and tracing the origins of parasites. The *PvMSP-1* gene is highly polymorphic and has been used as a marker in many *P. vivax* population studies. The aim of this study was to investigate the genetic diversity of the *PvMSP-1* gene icb5-6 fragment and to provide more genetic polymorphism data for further studies on *P. vivax* population structure and tracking of the origin of clinical cases.

**Methods:**

Nested PCR and sequencing of the *PvMSP-1* icb5-6 marker were performed to obtain the nucleotide sequences of 95 *P. vivax* isolates collected from Zhejiang province, China. To investigate the genetic diversity of *PvMSP-1*, the 95 nucleotide sequences of the *PvMSP-1* icb5-6 fragment were genotyped and analyzed using DnaSP v5, MEGA software.

**Results:**

The 95 *P. vivax* isolates collected from Zhejiang province were either indigenous cases or imported cases from different regions around the world. A total of 95 sequences ranging from 390 to 460 bp were obtained. The 95 sequences were genotyped into four allele-types (Sal I, Belem, R-III and R-IV) and 17 unique haplotypes. R-III and Sal I were the predominant allele-types. The haplotype diversity (Hd) and nucleotide diversity (Pi) were estimated to be 0.729 and 0.062, indicating that the *PvMSP-1* icb5-6 fragment had the highest level of polymorphism due to frequent recombination processes and single nucleotide polymorphism. The values of dN/dS and Tajima’s D both suggested neutral selection for the *PvMSP-1*icb5-6 fragment. In addition, a rare recombinant style of R-IV type was identified.

**Conclusions:**

This study presented high genetic diversity in the *PvMSP-1* marker among *P. vivax* strains from around the world. The genetic data is valuable for expanding the polymorphism information on *P. vivax*, which could be helpful for further study on population dynamics and tracking the origin of *P. vivax*.

**Electronic supplementary material:**

The online version of this article (doi:10.1186/s40249-017-0302-6) contains supplementary material, which is available to authorized users.

## Multilingual abstracts

Please see Additional file 1 for translations of the abstract into the five official working languages of the United Nations.

## Background

Malaria remains a severe public health problem worldwide, with more than 200 million cases causing 600 000–800 000 deaths annually [1]. About 9% of these cases are caused by the parasite *Plasmodium vivax* [1]. Although malaria induced by *P. falciparum* accounts for more deaths, *P. vivax* has a wider geographical distribution and a dormant liver stage which can easily lead to relapse.

Understanding the genetic diversity of malaria parasites from different regions is important for studies on population dynamics and is also valuable in discriminating parasite clones from infected individuals and tracing the origin of parasites [2, 3]. Currently, several genetic markers have been used to study *P. vivax* populations’genetic diversity [4–7], the most popular being *P. vivax* merozoite surface protein 1 (*PvMSP-1*). *PvMSP-1* is an important protein for erythrocyte invasion, and thus vaccine research, which is encoded by the *PvMSP-1* gene with approximately 1 720 amino acids. *PvMSP-1* is composed of conserved, partially conserved and variable regions. It has been shown in several studies that the *PvMSP-1* gene exhibits a mosaic-type structure due to recombination events [8–10]. As a partial fragment of the variable region in the *PvMSP-1* gene, the inter-species conserved fragments 5 (icb5) and icb6 show high polymorphisms from insertions, deletions, intra-allelic recombination and point mutations [4, 9–13]. The fragment consists of three pairs of dimorphic gene elements marked as I/II, P/Q and R/S, respectively. So far, four recombination types have been identified as Belem (I/Q/S), Salvador (Sal I, II/P/R)), recombination III (R-III, II/Q/S) and IV (R-IV, II/P/S) [8, 14, 15]. Compared to Sal I, Belem type contains a distinctive structure with a different length of Glutamine (Q) repeats. R-III and R-IV were the newly generated types through the recombination of Sal I and Belem. Since there is extensive genetic polymorphism of the *PvMSP-1* icb5-6 fragment, it is suitable for molecular discrimination of relapse episodes [8, 16], phylogenetic studies and genetic traceability studies [2, 17].

Most studies investigating the genetic diversity of *P. vivax* have focused on samples in a particular geographic region [6, 7, 12, 18]. Only a few studies were implemented to compare samples from different regions. In this study, *P. vivax* malaria samples from infections in different countries/regions (including Africa, South Asia, Southeast Asia, West Asia and partial regions of China) were collected in the Zhejiang province, aiming to investigate the genetic diversity of the *PvMSP-1* gene icb5-6 fragment and provide more genetic polymorphism data for further study on population structure and for tracking the origin of clinical cases.

## Materials and methods

### *S*ample collection

Blood samples were collected from indigenous and imported symptomatic *P. vivax* malaria patients in Zhejiang Province, China, from 2006 to 2013. The inclusion criteria were patients infected with *P. vivax* who were diagnosed definitely via microscopic examination and nested PCR. The microscopic examinations were done following the procedures of the WHO outlined in basic malaria microscopy [19], and the Nested PCR (NP) was implemented following the NP 1993 procedures [20]. All patients confirmed to have the *P. vivax* infection received normative anti-malarial drug treatment. An individual case survey was also conducted to find out whether they had a history of malaria and a travel history abroad. After informed consent from adults or legal guardians of children, 2.0 ml whole blood samples were collected in an anticoagulant tube and stored at −20 °C until DNA extraction was carried out.

### DNA extraction and species identification for *Plasmodium*

The total DNA was extracted from 200 μl of the whole blood using a QIAamp DNA Blood Mini Kit (QIAGEN, Germany), following the manufacturer’s instructions. DNA was eluted in 200 μl of Tris-EDTA buffer and kept at 4 °C until use, or stored at −20 °C. A standard nested PCR amplification method was used to identify *Plasmodium* species following previously reported protocols [20, 21]. The primers for nested PCR detection of *Plasmodium spp.* were listed in Additional file 2: Table S1. All amplification reactions were carried out in a total volume of 20 μl, containing 6.8 or 7.8 μl of ddH_2_O, 0.6 μl of each primer (10 μM), and 10 μl Green Master Mix (Promega, USA). Primary amplification reactions were initiated with the addition of 2.0 μl template genomic DNA prepared from the blood samples, and 1.0 μl of the primary reaction amplification was used as a template in the secondary amplification reactions. The amplified PCR products were visualized in 1.5% agarose gel containing 5 μl/100 ml GelRed Nucleic Acid Gel Stain (Biotium, USA).

### Amplification and sequencing of the *PvMSP-1* icb5-6 gene fragment

The DNA fragment of *PvMSP-1* icb5-6 was amplified by nested PCR following previously reported protocols [22]. The primers for nested PCR detection of *PvMSP-1* icb5-6 were listed in Additional file 2: Table S2. In brief, the primary PCR was performed in a total volume of 20 μl containing 2 μl DNA template, 10 μl PCR Green Master Mix (Promega, USA), 1 μl of each primer (10 μM), and 6 μl ddH_2_O. The secondary PCR was performed in a total volume of 50 μl containing 2 μl DNA template, 25 μl PCR Green Master Mix (Promega, USA), 2 μl of each primer (10 μM), and 19 μl ddH_2_O. The cycling parameters of the primary and secondary PCR were as follows: initial denaturation at 94 °C for 5 min, followed by 30 cycles of 94 °C for 1 min, 55 °C for 1 min, and 72 °C for 1 min, with a final extension of 72 °C for 5 min. The PCR products were visualized in 1.5% agarose gel electrophoresis containing 5 μl/100 ml GelRed Nucleic Acid Gel Stain (Biotium, USA). Positive PCR products were purified and directly sequenced in both directions using an ABI PRISM3730 DNA sequencer by Sangon Biotech (Shanghai, China).

### Data analysis

Nucleotide and amino acid sequences were aligned using MEGA version 6 with the following published sequences of Sal I (accession no. AF435602) and Belem (accession no. M60807) [23]. The *PvMSP-1* allele-types of *P. vivax* isolates were confirmed based on the alignment results. To find out the genetic diversity of the *PvMSP-1* icb5-6 fragment, some indices were determined using the DnaSP version 5, including the number and frequency of haplotypes, haplotype diversity (Hd), and the nucleotide diversity (π). In addition, the ratio of non-synonymous (dN) to synonymous (dS) substitution (dN/dS) was also calculated using DnaSP version 5 [24]. Additionally, to evaluate the neutral theory, Tajima’s D test was carried out through DnaSP version 5 [24]. In order to elucidate the genetic relationships among the isolates, the phylogenetic tree was constructed using the neighbor-joining method implemented by MEGA version 6.

## Results

### Sample distribution

In this study, 95 *P. vivax* samples were collected from Zhejiang province in Eastern China during 2006–2013. The *P. vivax* cases were mostly indigenous or imported from adjacent provinces during 2006–2011, while imported *P. vivax* cases from Southeast Asia, South Asia, etc*.* were dominant after 2012. From the 95 samples, sixty-nine showed a clear infection origin in the following areas: 2 in Africa (Ethiopia), 2 in Central China (Henan Province), 1 in Southwestern China (Yunnan Province), 39 in Eastern China (1 from Jiangsu Province, 30 from Anhui Province, 1 from Jiangxi Province, and 7 from Zhejiang Province), 6 in South Asia (India), 17 in Southeast Asia (6 from Myanmar, 1 from Indonesia, 8 from Cambodia, and 2 from Laos), and 2 in West Asia (Afghanistan).

### Allelic-type of *PvMSP-1* icb5-6 fragment

In this study, the *PvMSP-1* icb5-6 fragment was successfully amplified from the total parasitic DNA obtained from a single *P. vivax* infection. A total of 95 sequences ranging to 390 to 460 bp were obtained and aligned with the Sal I and Belem reference sequences. Based on three dimorphic elements, the 95 sequences were divided into four allele-types: Sal I (37, 38.94%), Belem (6, 6.32%), R-III (49, 51.58%) and R-IV (3, 3.36%). R-III and Sal I were the dominant allele-types. Sal I was mainly distributed in Southeast Asia, Africa, Eastern China, and South Asia. R-III was distributed in Southeast Asia, Eastern China, Central China, and West Asia. Belem was mainly distributed in Africa, South Asia, Southeast Asia, and Southwestern China, while R-IV was only found in Southeast Asia. The detailed distribution of the four allele-types is listed in Table 1.

**Table 1:** Geographical distribution of 95 *PvMSP-1* icb5-6 sequences based on allele-type level

Allele-type	Infection origin								Total
	Africa	Central China	Eastern China	South Asia	Southeast Asia	Southwestern China	West Asia	Unknown	
Belem	1	0	0	2	1	1	0	1	6
R-III	0	2	26	0	7	0	2	12	49
R-IV	0	0	0	0	2	0	0	1	3
Sal I	1	0	13	4	7	0	0	12	37
Total	2	2	39	6	17	1	2	26	95

The alignment of the 95 amino acid sequences revealed that Belem and R-III types have the structure of poly-Q, with the number of Q repeats ranging from 10 to 23. The poly-Q of Belem type mostly repeated more than 16 times, while it repeated mostly 10 times in R-III type, followed by 19 times, 18 times, 13 times and 23 times.

### Genetic diversity of the *PvMSP-1* icb5-6 fragment

During the alignment analysis, some gene gaps or missing data were found in partial sequences because of the differences in sequence length; therefore, only 330 bp gene sites were analyzed using DnaSP, and 65 SNPs (2 singleton variable sites and 63 parsimony informative sites) were found. As shown in Table 2, seventeen unique haplotypes were identified due to the occurrence of SNPs, with 12 haplotypes for Sal I, 2 haplotypes for Belem, 1 haplotype for R-III, and 2 haplotypes for R-IV. The haplotype diversity (Hd) and nucleotide diversity (Pi) were estimated to be 0.729 and 0.062, respectively. The analysis of synonymous and non-synonymous substitutions was performed for 106 codons of *PvMSP-1* icb5-6 fragments. The ratio of non-synonymous substitutions (dN) and synonymous substitutions (dS) were estimated to be 0.070 and 0.071, respectively. The dN/dS value was 0.986, which indicated that the majority of the polymorphic sites are changing under neutral selection. Tajima’s D test was also performed and estimated to be 1.873 (*P* > 0.05), which indicates neutral selection for the *PvMSP-1* protein.

**Table 2:** The allelic-type and frequencies of nineteen haplotypes

Haplotype	Isolates No.	Allelic-type	Haplotype	Isolates no.	Allellic-type
Hap_1*	1	Sal I	Hap_11	5	Sal I
Hap_2	2	Sal I	Hap_12	4	Belem
Hap_3	1	Sal I	Hap_13	2	Belem
Hap_4	8	Sal I	Hap_14	3	Sal I
Hap_5	2	R-IV	Hap_15	1	Sal I
Hap_6	8	Sal I	Hap_16	1	Sal I
Hap_7	4	Sal I	Hap_17	2	Sal I
Hap_8	1	Sal I	Hap_18	1	Sal I
Hap_9	1	R-IV	Hap_19*	1	Belem
Hap_10	49	R-III	-	-	-

*:The asterisk represent the reference sequences Sal I (AF435602) and Belem (M60807)

**Fig. 1:**
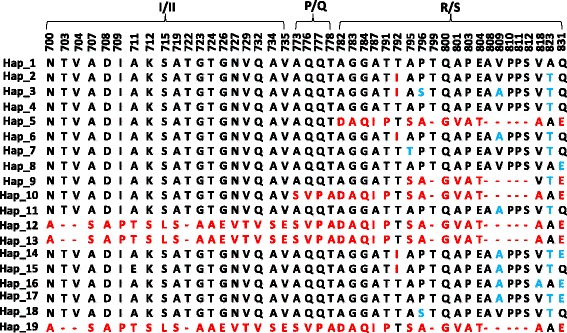
Sequence polymorphisms of *PvMSP-1* icb5-6 amino acids identical to those of the reference strain, Sal I (AF435602, protein_id: AAN86215.1). Amino acids identical to those of the reference strain are marked in *black* typeface, the substituted amino acids are marked in *red* and *blue* typeface

**Fig. 2:**
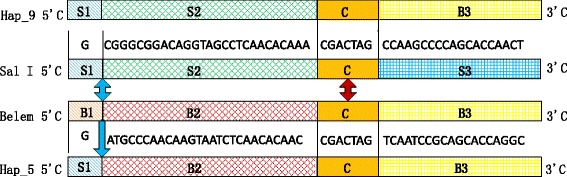
The putative recombinant sites between P/Q and R/S elements of Hap_5 and Hap_9. (*Blue arrow*: recombinant site of Hap_5; *Red arrow*: recombinant site of Hap_9)

A typical sequence of 17 haplotypes was randomly selected for *PvMSP-1*icb5-6 amino acids sequence polymorphisms analysis. Forty-four amino acids polymorphism sites are shown in Fig. 1. Hap_1 and Hap_19 were the reference sequences for the Sal I and Belem, respectively. Seven non-synonymous changes at codon T792I, A795T, P796S, V809A, V818A, A823T, and Q831E were detected in the 12 haplotypes for Sal I type. There were no non-synonymous changes among the two haplotypes for Belem type (Hap_12 and Hap_13) and Hap_19. Hap_10 was the only haplotype for R-III in which the polymorphism sites were Sal I-like in the element of I/II and Belem-like in the elements of P/Q and R/S. Hap_5 and Hap_9 were the haplotypes for R-IV in which the polymorphism sites were Sal I-like in the elements of I/II and P/Q and Belem-like in the element of R/S. However, the amino acids at codon 782–784, 787, 791/818, and 823 of Hap_9 were AGGAT/VT in the R/S element, which was the same as Sal I but not the same as Belem. The polymorphism between Hap_5 and Hap_9 indicated that the recombinant sites between P/Q and R/S elements were different. The two putative recombinant sites were marked in Fig. 2. As shown in the figure, in the region of P/Q and R/S elements, the sequences of Sal I and Belem were separated into the four regions of S1, S2, C, and S3, and B1, B2, C, and B3, respectively; C was the conservative region. The recombinant site normally located between S1 and B2, and Hap_5 was generated with the structure of S1, B2, C, and B3. However, Hap_9 was generated with the structure of S1, S2, C, and B3, due to the putative recombinant site located in the C region.

### Sequence polymorphism and clustering of *PvMSP-1*

**Fig. 3:**
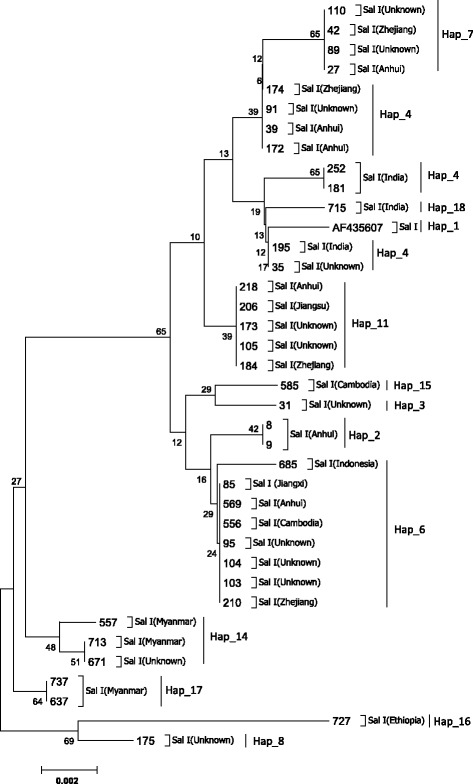
Dendrogram of the *PvMSP-1* icb5-6 based on the nucleotide sequences of 37 isolates of Sal I type and Sal I reference sequence (AF435602)

**Fig. 4:**
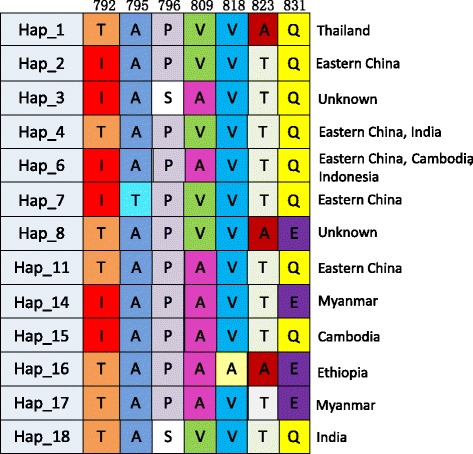
The molecular barcode for haplotypes of Sal I type. Hap_1 represent the Sal I reference strain (AF435602, protein_id: AAN86215.1)

**Fig. 5:**
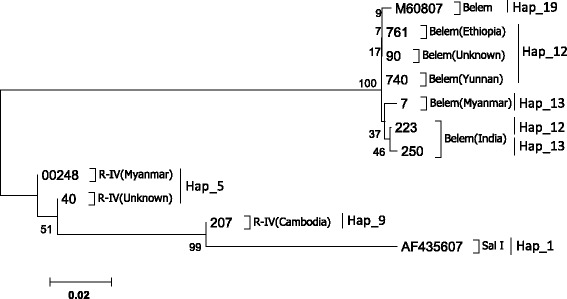
Dendrogram of the *PvMSP-1* icb5-6 based on the nucleotide sequences of 6 isolates of Belem type, 3 isolates of R-IV type and Sal I reference sequence (AF435602), Belem reference sequence (M60807)

Among the four allele-types, Sal I showed more diversity than the other three types. A dendrogram of *PvMSP-1* icb5-6 sequences was constructed using 37 nucleotide sequences from Sal I type. This analysis grouped the 37 isolates into multi-subtypes with the corresponding haplotype (Fig. 3). The dendrogram showed some significant relationships between subtype and infection source, such as the five subtypes (Hap_4, 7, 11, 2, 6) in Eastern China, two subtypes (Hap_4, 18) in India, and the subtype (Hap_14) which was unique to Myanmar. Using the sequence polymorphisms of *PvMSP-1* icb5-6 amino acids shown in Fig. 1, the relationship between amino acid sequence polymorphisms and their origin has been summarized for *P. vivax* tracking (Fig. 4). For example, according to the barcode and dendrogram, the isolates coding 173 and 105 were predicted to come from Eastern China, while the sample coding 671 may have come from Myanmar. The dendrogram of *PvMSP-1* icb5-6 sequences was also constructed using 6 nucleotide sequences from Belem type and 3 nucleotide sequences from R-IV type (Fig. 5). Belem and R-IV were both grouped into two haplotypes, which were less diverse than Sal I type. Forty-nine isolates of R-III were grouped into one haplotype- it was highly conservative for the isolates from different regions.

### Discussion

The malarial parasite *P. vivax* was eradicated in many subtropical countries and significantly reduced in some tropical regions through the application of vector control measures and the deployment of mass treatment of febrile individuals. Unfortunately, climate change, socioeconomic change and human population movements have contributed to the re-emergence of the infection in some malaria-free areas. Therefore, *P. vivax* remains a potential cause of morbidity and mortality for the people living in endemic areas. In malaria hypo-endemic regions, different parasite genotypes are circulating and geographic isolation may exist [5]. Additionally, *P. vivax* populations and transmission patterns are also changing constantly with increasing movement of human populations [25].

To better understand *P. vivax* populations and transmission dynamics, the genetic diversity of *P. vivax* was investigated using the genetic marker *PvMSP-1*. In this study, 95 *P. vivax* isolates were collected from the Zhejiang province of Eastern China during 2006–2013. Zhejiang province was once a low epidemic area of *vivax* malaria. The last local malaria case was reported in 2011. Although Zhejiang province promised to achieve the goal of eliminating malaria by 2016, malaria importation remains a serious challenge because a vector (*Anopheles sinensis*) still exists, and with imported cases could cause a resurgence in susceptible areas.

The *PvMSP-1* gene exhibits high genetic variation and complex structuring in a number of geographic regions around the world [9]. It has been used as marker in many genetic studies of *P. vivax* [4, 12, 26]. Mutation and frequent recombination events are the main mechanisms responsible for the high genetic diversity observed in the *P. vivax* icb5-6 fragment [9]. Four allele-types, Belem, Sal I, and recombination types (R-III and R-IV), of the *PvMSP-1* icb5-6 fragment have been described worldwide [8, 14]. In the present study, the four allele-types were identified from 95 *P. vivax* isolates. Because of quantitative limitations and geographic restriction of *P. vivax* sampling, this study was limited in its ability to describe the diversity of *PvMSP-1* icb5-6 around the world. However, among the cases with a clear infection origin, over half of cases (56.5%) were distributed in Eastern China, followed with 24.6% in Southeast Asia, 8.7% in South Asia, 2.9% in Central China, 2.9% in Africa, 2.9% in West Asia and 1.4% in Southwestern China. R-III was the dominant type in Eastern China, followed by Sal I, while Belem and R-IV were absent in this region. This result was in concordance with previous reports in the Anhui province (the other province of Eastern China) [18]. It has been reported that the generation of R-types arise from intragenic recombination of Belem and Sal I types in mosquito vectors [9, 27]. The dominance of R-III type with the absence of Belem type in Eastern China made the generation of the R-III type unusual, which could be related to sampling bias, population changes of mosquito species, or selection by host immunity pressure on specific types of parasite. In fact, studies conducted in Southern Mexico reported that the distribution of different *P. vivax* populations was largely determined by their infectivity of two species of anopheline vectors [28]. Hence, in Eastern China, the changes in mosquito strains may lead to changes in parasite population structure. The four allele-types were all detected in Southeast Asia, with Sal I and R-III being the dominant allele-types. The population structure of *P. vivax* in Southeast Asia was more diverse than in other regions. The high levels of recombinant types and Sal I in Southeast Asia are in concordance with the report from Myanmar [26]. Besides this, Southeast Asia was the only region where R-IV was detected. The Belem type was also observed in Southwestern China, and it can be speculated that this region may have a similar allele-type distribution to Southeast Asia, because it was located on the border between China and countries of Southeast Asia.

In the present study, the analysis of single-nucleotide polymorphisms revealed 63 parsimony informative sites and a total of 17 haplotypes were observed based on the SNPs. Values of dN/dS and Tajima's D both suggested neutral selection for the region of *PvMSP-1* icb5-6, a result which differs compared to a study conducted in Southern Mexico, where the dN/dS test indicated that different icb5-6 subfragments might be subjected to either positive or purifying selection [29]. This difference may have resulted from sampling bias or differences in analysis structure, as the analysis in the present study was based on the level of the entire icb5-6 fragment, while it was analyzed by each subfragment in the Mexican study.

In this study, Sal I type consisted of 12 haplotypes and it was more diverse than the other three allele-types. Based on the polymorphism sites, the relationship between amino acid sequence polymorphisms and case origin was summarized for the preliminary identification and tracking of *P. vivax*. R-IV was relatively rare and was only detected in Southeast Asia. It consisted of two haplotypes, Hap_5 and Hap_9. The amino acids of Hap_9 at codon 782–784,787,791/818, and 823 were AGGAT/VT, which were the same as Sal I, while those of Hap_5 were DAQIP/AA, which were the same as Belem. The recombinant site of Hap_5 was the most common recombination type, which is the same as previously reported from other *vivax* malaria endemic regions around the world. Conversely, Hap_9 was identified as a unique variant, generated via a rare recombination site which was also identified in a previous study [4].

## Conclusion

In this study, the genetic diversity and phylogenetic relationship of the *PvMSP-1* gene icb5-6 fragment was analyzed. Four allelic-types of *PvMSP-1* and seventeen haplotypes were identified. Besides this, a rare recombinant site was detected in Hap_9. Based on the genetic diversity of Sal I type, a molecular barcode was proposed for preliminary tracking of *P. vivax*. The information on the genetic diversity of the *PvMSP-1* icb5-6 fragment presented in this study provides positive evidence for genetic recombination in *P. vivax* populations and a basis for the correct classification and tracking of *P. vivax* cases.

## Additional files

Additional file 1: Multilingual abstracts in the five official working languages of the United Nations. (PDF 1068 kb)

Additional file 2: The primers for nested PCR detection of *Plasmodium spp.* and *PvMSP-1*. (DOCX 14 kb)

## Abbreviations

°C: Celsius
icb: Inter-species conserved fragment
min: Minute
ml: Milliliter
No.: Number.
*P. vivax*: *Plasmodium vivax*
*PvMSP-1*: *P. vivax* merozoite surface protein-1
Q: Glutamine
R-III: Recombination III
R-IV: Recombination IV
Salvador: SalI
SNP: Single nucleotide polymorphism
μl: Microliter
μM: Micromolar
