# Supplementary material for: Genetic diversity of Plasmodium Vivax revealed by the merozoite surface protein-1 icb5-6 fragment
Source: Infect Dis Poverty. 2017 Jun 5;6:92. doi: 10.1186/s40249-017-0302-6 (PMC5458480; doi:10.1186/s40249-017-0302-6)
Supplement: Supplementary file 1 — Multilingual abstracts in the five official working languages of the United Nations. (PDF 1068 kb) [file 40249_2017_302_MOESM1_ESM.pdf]

Translation of the abstract into the five official working languages of the United Nations

**كشف التنوع الجيني للمتصورة النشيطة من خلال قسيمة الملاريا للبروتين السطحي-1 الشُدفة icb5-6**


وي روان، لينج لينج تشانج يان فنج، شوان تشانج هوا ليانج تشن تشياو يي لو، لي-نونج ياو وى هو جين تاو



**ملخص**

**خلفية:** لا تزال المتصورة النشيطة سببا محتملا للمراضة والوفيات للأشخاص الذين يعيشون في المناطق في المستوطنة. يعد فهم التنوع الجيني للمتصورة النشيطة في مختلف المناطق أمرا هاما في دراسة الديناميات السكانية وتتبع أصول الطفيليات. الجين PvMSP-1 متعدد الأشكال للغاية ويستخدم كعلامة في العديد من الدراسات السكانية للمتصورة النشيطة. وكان الهدف من هذه الدراسة التعرف على التنوع الجيني للشُدفة icb5-6 في الجين PvMSP-1 وتقديم المزيد من بيانات تعدد الأشكال الوراثية لمزيد من الدراسات للتركيبة السكانية المتصورة النشيطة وتتبع أصل الحالات السريرية.

**الطرق:** أجريت تفاعلات البلمرة التسلسلية وتسلسل الشدفة PvMSP-1 icb5-6 للحصول على تسلسل النوكليوتيدات من 95 من المتصورة النشيطة المعزولة من مقاطعة تشجيانج في الصين. للتحقيق في التنوع الجيني لـPvMSP-1 ، تم ترميز تسلسل 95 من النوكليوتيدات من الشدفة PvMSP-1 icb5-6 وتحليلها باستخدام DnaSP v5 من برنامج MEGA.

**النتائج:** 95 من المتصورة النشيطة المعزولة المجلوبة من مقاطعة تشجيانج إما حالات أصلية أو حالات وافدة من مناطق مختلفة حول العالم. وقد تم الحصول على ما مجموعه 95 متوالية تتراوح 390-460 نقطة أساس. تم التنميط الجيني لتسلسل 95 إلى أربعة أنواع أليل (Sal I، Belem، R-III، R-IV) و17 نمطا فردانيا فريدا من نوعه. كان الأليل R-III وSal I هو السائد. وقدرت تنوع النمط الفرداني (HD) والتنوع النوكليوتيدات (Pi) ليكون 0.729 و0.062، مشيرا إلى أن الشدفة PvMSP-1 icb5-6 بها أعلى مستوى من تعدد الأشكال نظرا لعمليات إعادة التركيب المتكررة وأحادية تعدد أشكال النوكليوتيدات. وأظهرت قيم DN / DS واختبار تاجيما D على حد سواء الاختيار المحايد للشدفة PvMSP-1icb5-6. وبالإضافة إلى ذلك، تم تحديد أسلوب مؤتلف نادر لنوع R-IV.

**الاستنتاجات:** أظهرت هذه الدراسة وجود تنوع جيني عال في الواسم الجيني PvMSP-1 بين سلالات المتصورة النشيطة من جميع أنحاء العالم. وتعد البيانات الوراثية قيمة لتوسيع المعلومات حول تعدد أشكال المتصورة النشيطة، التي يمكن أن تكون مفيدة عن إجراء مزيد من الدراسة على الديناميات السكانية وتتبع أصل المتصورة النشيطة.


Translated from English version into Arabic by Mahmoud Sami, through

TRANSLATORS
WITHOUT BORDERS

**间日疟原虫裂殖子表面蛋白 1（*MSP-1*） icb5-6 区段基因多态性分析**


阮卫，张玲玲，丰燕，张轩，陈华良，陆巧绎，姚立农，胡薇



**摘要**

**引言：**间日疟原虫仍是引起疟疾流行区居民发病和死亡的一个潜在病因。了解不同地区间日疟原虫的基因多态性对种群动力学研究以及虫体溯源具有重要意义。*PvMSP-1* 基因具有高度多态性，作为分子标记物已被广泛应用于间日疟原虫种群研究中。本研究的目的在于进一步探索 *PvMSP-1* 基因的多态性，为间日疟原虫的种群结构研究和临床疟疾病例的溯源提供更多的依据。

**方法：**选取从中国浙江省发现的已确诊间日疟原虫 95 例作为研究样本，应用巢式 PCR 和分子测序的方法获取 *PvMSP-1* icb5-6 区段的核苷酸序列，应用 DnaSP v5, MEGA 等生物信息学软件

对 95 条序列进行分析，获取该区段基因的多态性信息。
**结果：**95 例间日疟原虫病例分别为国内本土病例和来自世界不同地区的输入性病例。共获得核苷酸长度约为 390-460 bp 的测序序列 95 条。95 条序列分为 4 种等位基因型（Sal Ⅰ, Belem, R-III 和 R-Ⅳ）和 17 种单倍型，其中 R-III 和 Sal Ⅰ是主导的等位基因型。单倍型多样性（Hd）和核苷酸多样性（Pi）值分别为 0.729 和 0.062，表明 *PvMSP-1* 基因 icb5-6 区段由于存在多个重组现象和单核苷酸多态，具有较高水平的多态性。dN/dS 和 Tajima’s D 值均表明 *PvMSP-1* 基因在中性选择下进化。此外，在 R-Ⅳ等位基因型中发现一种较为罕见的重组类型。
**结论：**本研究以来自世界不同地区的间日疟原虫虫株作为研究样本，探索了间日疟原虫 *PvMSP-1* 基因的多态性特征，该多态性信息丰富了间日疟原虫的基因多态性数据，有助于种群动力学的进一步研究和间日疟原虫的溯源研究。

Translated from English version into Chinese by Ling-ling Zhang.

## Diversité génétique du *Plasmodium vivax* révélée par un fragment de protéine de surface de mérozoïte -1 (PvMSP-1) icb5-6

Wei Ruan, Ling-ling Zhang, Yan Feng, Xuan Zhang, Hua-liang Chen, Qiao-yi Lu, Li-nong Yao, Wei Hu

**Résumé**
**Contexte:** Le *Plasmodium vivax* reste une potentielle cause de morbidité et de mortalité pour les populations qui vivent dans des zones endémiques. La compréhension de la diversité génétique du *Plasmodium vivax* de plusieurs régions variées est essentiel à l'étude de la dynamique des populations et à la recherche des origines des parasites. Le gêne *PvMSP-1* est très polymorphe et a très souvent été utilisé comme marqueur dans plusieurs études sur les populations de *P. vivax.* Cette étude vise à déterminer la diversité génétique du fragment du gêne *PvMSP -1 icb5-6* apporter plus de données sur le polymorphisme génétique pour des études plus approfondies sur la structure de la population du *P. vivax* et la recherche de l'origine des cas cliniques.
**Méthodologie:** Des tests de nested PCR et un séquençage du marqueur *PvMSP-1* ont été administrés pour obtenir les séquences de nucléotides de 95 *P. vivax* isolés que nous avons prélevés dans la province de Zhejiang en Chine. Pour étudier la diversité génétique du *PvMSP-1*, le génotypage des 95 séquences de nucléotide du fragment *PvMSP-1 icb5-6* a été établi et analysé à l'aide du logiciel DnaSPv5, MEGA.
**Résultats:** Les 95 *P. vivax* isolés que nous avons prélevés dans la province de Zhejiang étaint constitués de cas propres à la régions et de cas importé de d'autres régions du monde. Au total, une pair de base s'étendant de 390 à 460 pour les 95 séquences a été obtenue. Le génotypage des 95 séquences s'est fait en quatre types d'allèles (Sall, Belem, R-III et R-IV) dont 17 haplotypes uniques. R-III et Sall étaient les types d'allèles dominants. La diversité des haplotypes (Hd) et la diversité des nucléotides (Pi) étaient estimées à 0,729 et 0,062 du fait du taux le plus élevé de polymorphisme du fragment du *PvMSP-1 icb5-6* à cause des nombreux processus de recombinaison et des polymorphismes de chaque nucléotide. Les valeurs dN/dS et du D Tajima ont toutes suggéré une sélection neutre pour le fragment *PvMSP-1 icb5-6*. Par ailleurs, une recombinaison aussi rare que celle de type R-IV a été identifiée.

**Conclusions:** Cette étude a démontré que des souches du *P. vivax* du monde entier, le marqueur *PvMSP-1* a une grande diversité génétique. En effet, les données génétiques seront utile à accroître les informations sur le *P. vivax,* ce qui pourrait nous orienter des recherches futures sur la dynamique des populations et la recherche des origines du *P. vivax.*

Translated from English version into French by simonyetna, through

**Генетическое разнообразие Малярийных Плазмодий, выявленных мерозоитом поверхностного белка-1 и фрагментом icb5-6**

Wei Ruan, Ling-ling Zhang, Yan Feng, Xuan Zhang, Hua-liang Chen, Qiao-yi Lu, Li-nong Yao, Wei Hu

**Реферат**

**Фон:** Малярийные Плазмодии остаются потенциальной причиной заболеваемости и смертности среди людей, живущих в эндемичных районах. Понимание генетического разнообразия *P. vivax* из разных регионов представляет ценность для изучения динамики численности и прослеживания происхождения паразитов. Ген *PvMSP-1* является высоким полиморфизмом и был использован в качестве маркера во многих исследованиях по *P. vivax* населениям. Целью данного исследования было изучение генетического разнообразия *PvMSP-1* ген icb5-6 фрагмент и обеспечить более генетических данных полиморфизма для дальнейших исследований по *P. Vivax* структура населения и отслеживании происхождения клинических случаев.

**Методы**: Гнездовой PCR и секвенирования *PvMSP-1* icb5-6 маркеров были выполнены для получения нуклеотидных последовательностей 95 *P.* vivax изолятов, собранных из провинции Чжэцзян, Китай. Для изучения генетического разнообразия PvMSP-1, в 95 нуклеотидных последовательностей *PvMSP-1* icb5-6 фрагмент были генотипированы и проанализированы с помощью DnaSP у5, программным обеспечением МЕГА.

**Результаты**: 95 *P.* vivax изолятов, собранных из провинции Чжэцзян были либо коренные жители, либо завозные случаи из разных регионов мира. Были получены в общей сложности 95 последовательностей в диапазоне от 390 до 460 bp. 95 последовательностей были генотипированы на четыре аллеля-типов (Sal I, Belem, R-III- и R-IV) и 17 уникальных гаплотипов. R-III и Sal Iбыли преобладающими аллель-типами. Разнообразие гаплотипа (Hd) и нуклеотидное разнообразие (Pi) оценивались в 0.729 и 0.062, указывая на то, что *PvMSP-1* icb5-6 фрагмент был самый высокий уровень полиморфизма в связи с частыми процессами рекомбинации и однонуклеотидным полиморфизмом. Значения dN/dS и Д Тадзима предложили нейтральный выбор для *PvMSP*-1icb5-6 фрагмент. Кроме того, был выявлен редкий рекомбинантный стиль по типу R-IV.

**Заключение**: Данное исследование представляет высокое генетическое разнообразие в *PvMSP-1* маркер между штаммами *P. vivax* во всем мире. Генетические данные важны для расширения информационного полиморфизма на *P. vivax*, которые могут быть полезными для дальнейшего

изучения динамики населения и отслеживания происхождения *P. vivax*.

Translated from English version into Russian by Hao-Qi Zhang

**Diversidad genética del *Plasmodium vivax* revelada por un segmento de la proteína 1 de superficie de merozoito ICB5-6**

Wei Ruan, Ling-ling Zhang, Yan Feng, Xuan Zhang, Hua-liang Chen, Qiao-yi Lu, Li-nong Yao, Wei Hu

**Reseña**

**Antecedentes**: el *Plasmodium vivax* todavía es una causa potencial de morbilidad y mortalidad para las personas que viven en áreas endémicas. Comprender la diversidad genética del *P. vivax* de regiones diferentes es valioso para estudiar la dinámica de la población y rastrear los orígenes de parásitos. El gen *PvMSP-1* es altamente polimórfico y se ha usado como marcador en muchos estudios de población del *P. vivax*. El objetivo de este estudio era investigar la diversidad genética del segmento ICB5-6 del gen *PvMSP-1* y proveer más datos de polimorfismo genético para estudios más extensos sobre la estructura de la población del *P. vivax* y rastrear el origen de casos clínicos.

**Métodos**: se llevaron a cabo la PCR anidada y la secuenciación del marcador *PvMSP-1* icb5-6 para obtener las secuencias nucleotídicas de 95 aislados de *P. vivax* recolectados de la provincia de Zhejiang, China. Para investigar la diversidad genética de *PvMSP-1*, se determinaron los genotipos de 95 secuencias nucleotídicas del segmento icb5-6 de *PvMSP-1* y se analizaron usando software DnaSP v5 y MEGA.

**Resultados:** Los 95 aislados de *P. vivax* recolectados de la provincia de Zhejiang eran o casos nativos o casos importados de diferentes regiones del mundo. Se obtuvo un total de 95 secuencias que iban desde 390 a 460 bp. Se determinó la genotipificación de 95 secuencias en cuatro tipos de alelos (SalI, Belem, R3 y R4) y 17 haplotipos únicos. R3 y SalI eran los tipos de alelos predominantes. La diversidad haplotípica (Hd) y la diversidad nucleotídica (Pi) se estimaron en 0.729 y 0.062, lo que indicaba que el segmento ICB5-6 de *PvMSP-1* tenía el nivel más alto de polimorfismo debido a los frecuentes procesos de recombinación y polimorfismo de un solo nucleótido. Tanto los valores de dN/dS como la D de Tajima sugirieron la selección neutral para el segmento *PvMSP-1*icb5-6. Además, se identificó un recombinante raro de tipo R4.

**Conclusiones**: Este estudio presentó una diversidad genética alta en el marcador *PvMSP-1* entre las cepas del *P. vivax* de alrededor del mundo. Los datos genéticos son valiosos para ampliar la información de polimorfismo en el *P. vivax*, lo que podría ser útil para estudios más extensos sobre la dinámica de la población y rastrear el origen del *P. vivax*.

Translated from English version into Spanish by LidiaN, through

TRANSLATORS
WITHOUT BORDERS
