# Supplementary material for: Genetic diversity of Plasmodium Vivax revealed by the merozoite surface protein-1 icb5-6 fragment
Source: Infect Dis Poverty. 2017 Jun 5;6:92. doi: 10.1186/s40249-017-0302-6 (PMC5458480; doi:10.1186/s40249-017-0302-6)
Supplement: Supplementary file 2 — The primers for nested PCR detection of Plasmodium spp. and PvMSP-1. (DOCX 14 kb) [file 40249_2017_302_MOESM2_ESM.docx]

Table S1 Primers for nested PCR detection of *Plasmodium* spp.

| Primer name | Primer sequence（5’→3’） | *Plasmodium* spp. | Product/bp |
| --- | --- | --- | --- |
| rPLU5 | CCTGTTGTTGCCTTAAACTTC | *Plasmodium* spp. | 1 200 |
| rPLU6 | TTAAAATTGTTGCAGTTAAAACG |  |  |
| rFAL1 | TTAAACTGGTTTGGGAAAACCAAATATATT | *P. falciparum* | 205 |
| rFAL2 | ACACAATGAACTCAATCATGACTACCCGTC |  |  |
| rVIV1 | CGCTTCTAGCTTAATCCACATAACTGATAC | *P. vivax* | 120 |
| rVIV2 | ACTTCCAAGCCGAAGCAAAGAAAGTCCTTA |  |  |
| rMAL1 | ATAACATAGTTGTACGTTAAGAATAACCGC | *P. malariae* | 144 |
| rMAL2 | AAAATTCCCATGCATAAAAAATTATACAAA |  |  |
| rOVA1 | ATCTCTTTTGCTATTTTTTAGTATTGGAGA | *P. ovale curtisi* | 800 |
| rOVA2 | GGAAAAGGACACATTAATTGTATCCTAGTG |  |  |
| rOVA1v | ATCTCCTTTACTTTTTGTACTGGAGA | *P. ovale wallikeri* | 780 |
| rOVA2v | GGAAAAGGACACTATAATGTATCCTAATA |  |  |

Table S2 Primers for nested PCR of *PvMSP-1icb5-6*

| Primer name | Primer sequence（5’→3’） |
| --- | --- |
| Pvmsp1-F1 | CCCTACTACTTGATGGTCCTC |
| Pvmsp1-R1 | CCTTCTGGTACAGCTCAATG |
| Pvmsp1-F2 | AGCATGATCGCCACTGAGAAG |
| Pvmsp1-R2 | GTGCTTGTGACATGCGTAAGC |
